# Invisible doppelgänger and body image disorders in right superior parietal lobule stroke, a case series

**DOI:** 10.1002/ibra.12057

**Published:** 2022-08-08

**Authors:** Emre Kumral, Fatma E. Çetin, Birgül Dere, Hüseyin N. Özdemir

**Affiliations:** ^1^ Neurology Department, Medical School Hospital Ege University İzmir Turkey; ^2^ Neurology Department Acıbadem Hospital Bursa Turkey

**Keywords:** asomatognosia, autoscopic phenomena, fading limb, invisible doppelgänger, superior parietal lobule

## Abstract

Autoscopic phenomena or an “invisible doppelgänger” refer to the illusory reduplication of one's own body. Body image disorder involves distorted perception or decreased body awareness. In the literature, feeling of presence (FOP) is rarely presented with a circumscribed cerebral pathology due to acute vascular lesions, and concomitant FOP and disorders of the body image or the body schema (BIBS) have rarely been reported. We present three cases of both FOP and BIBS disorders. All patients reported the two types of symptoms almost simultaneously: The first patient had the symptom of somatoparaphrenia characterized by deny ownership of the hand and feeling that it does not belong to her, the second patient had the sign of fading limb presented with misuse of his left hand when it was not under visual control and could not mentally represent and locate this part of the body in space, and the third patient had autotopagnosia; he was unable to localize any touched area below the elbow and knee. All patients had right parietal ischemic lesions involving the superior parietal lobule, and two patients had an adjacent additional precuneal involvement. Based on the cases presented here, it is plausible that BIBS may develop in addition to FOP, especially in lesions involving the superior parietal lobule and precuneus.

## Graphical abstract

This paper describes three patients who had both the feeling of presence (FOP) and body image or body schema disorders. All patients reported almost simultaneously the invisible doppelgänger presence and at least one sense of body schema and image disorder. In terms of these cases, it is possible that body image and schema disorders may have developed in addition to FOP, especially in lesions involving the superior parietal lobule and precuneus.

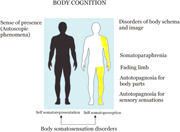

## 1 INTRODUCTION

The autoscopic phenomena encompass a wide range of experiences, including the deceptive reduplication of one's own body.^1^ The sense of a presence (FOP) refers to awareness of another person's physical presence or the sense of a “presence” in or near the extrabody space.^1^, ^2^ A “double” or an “invisible doppelgänger,” a more or less exact replica of one's body, is seen or felt in the personal space, or observed from a seemingly disjointed perspective of one's body. Disorders of the body image or the body schema (BIBS) can include distorted perception or decreased awareness of parts of the body or impaired ability to localize them (autotopagnosia), feelings of strangeness, fading limb, and illusory feelings of movement or even of having supernumerary limbs.^3^, ^4^, ^5^ Although it is accepted that BIBS disorders may be associated with additional syndromes in parietal lobe lesions, the reasons for their presence or absence in individual patients have often been a mystery.

While lateralization of lesions in the invisible doppelgänger remains controversial,^6^, ^7^ the deficiencies that cause BIBS disorders are usually associated with syndromes that occur as a result of parietal lobe lesions, although they may also occur following lesions associated with the temporal or frontal lobes. While FOP does not develop solely due to occipital lobe lesions, parietal, frontal, insular, and temporal‐limbic structures have also been reported to be involved.^6^, ^8^, ^9^ Acausal link between this type of doppelgänger phenomenon and various vestibular dysfunctions has often been suggested.^10^ FOP has been reported in subjects during long periods of social isolation and in many focal or diffuse brain diseases: psychiatric disorders, head trauma, tumors, brain infectious or inflammatory diseases, epilepsy, migraine, and degenerative diseases. Contrary to BIBS disorders, which are often caused by cerebrovascular lesions,^4^ the “invisible doppelgänger” is rarely associated with cerebral‐strokes.

In this case series, we report three patients who had both the FOP and BIBS disorder due to right superior parietal and precuneal ischemic stroke, and the underlying anatomy was determined using magnetic resonance imaging (MRI).

## 2 METHODS

Between 2010 and 2020, 4400 patients with ischemic stroke were admitted to the Stroke Unit. A total of 116 patients with MRI‐proven ischemic lesions restricted to the parietal cortex were identified. MRI examination was performed within the first week after admission. Axial T1‐, T2‐weighted spin‐echo, diffusion‐weighted image (DWI), and fluid attenuation inversion recovery sequences were obtained. Axial MRI T2‐weighted or DWI axial cuts of the scans were reviewed in a consensus fashion to determine the presence of lesions based on visual inspection. Clinical and neuropsychological signs and symptoms of invisible doppelgänger and BIBS were evaluated according to previous descriptions.^4^, ^7^, ^8^ None of the patients had a history of prestroke dementia, delirium, Parkinson's disease, seizure disorders, depression, psychosis, anxiety disorder, use of psychotropic prescription or nonprescription drugs, and history of visual and auditory hallucinations. Written informed consent was obtained from patients for publication of this case series and accompanying images (approval number: 22‐5T/59).

### 2.1 CASE 1

A 62‐year‐old right‐handed woman with no neurological or psychiatric history had a strong feeling that her left arm was changed when she awoke and felt that the limb did not belong to her, as if it was alien to her. After examining her left hand in amazement, she realized that her left hand was her own, but for a few moments, her left arm became alienated again and she could not control it for a while. After this situation continued for half an hour, she felt that there was a presence on her left in the kitchen. She felt that it was a “person,” moving and touching her shoulder. After she turned her head, the belief that someone else was there immediately returned, and she suddenly felt that this “person” was herself, the “Doppelgänger.” She saw that the woman's face was just like hers. She could identify the face and the upper part of the body including the color of the dress and the expression of the face. The image always appeared to the left side and in front of her.

**Figure 1:**
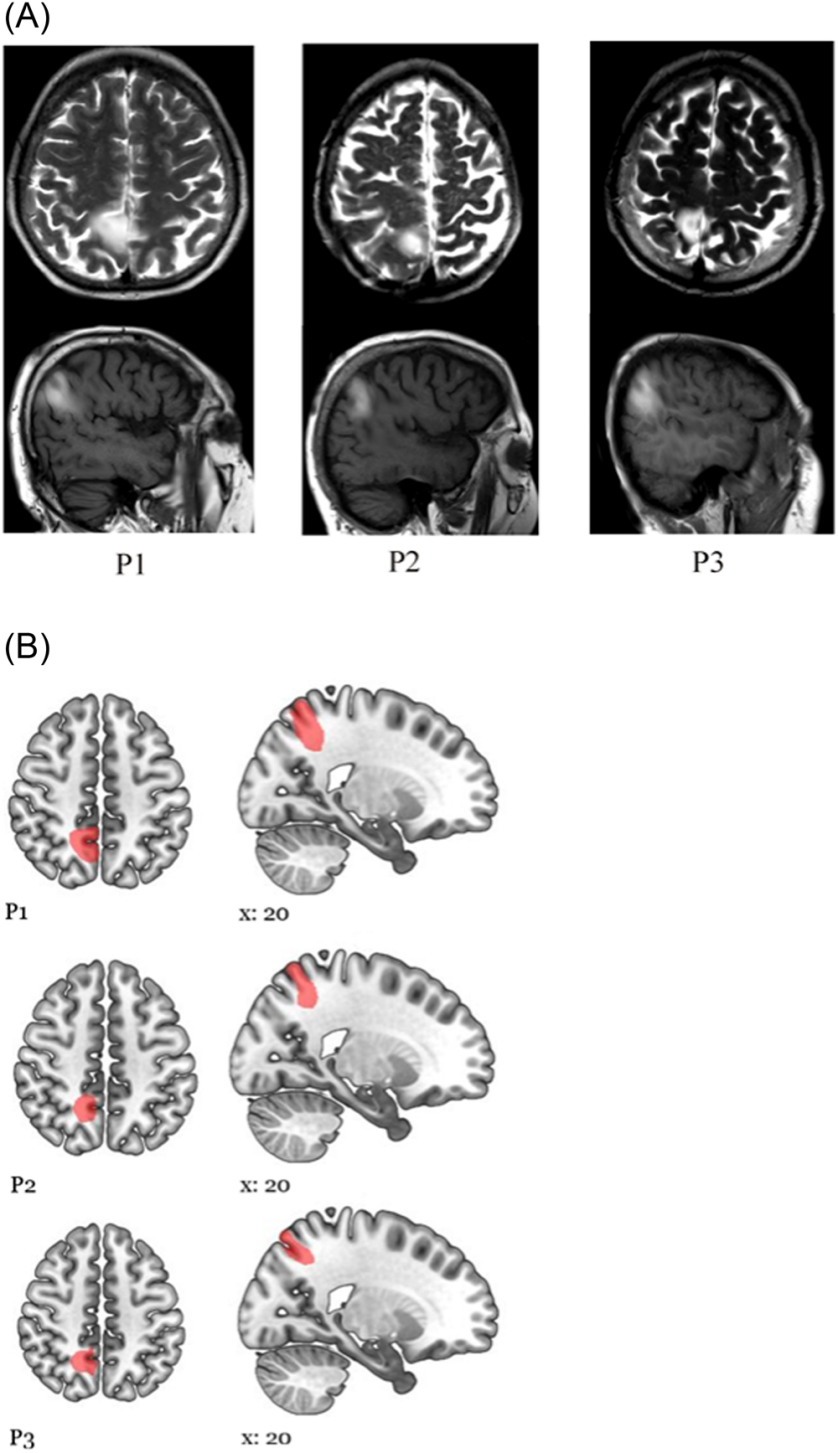
(A) Brain MRI images of patients obtained within 1 week after stroke. Axial T2‐weighted and coronal FLAIR images showed ischemic lesions in the right superior parietal lobule and the precuneal cortex. (B) Schematic drawing of the lesions in each patient. FLAIR, fluid attenuation inversion recovery; MRI, magnetic resonance imaging. [Color figure can be viewed at wileyonlinelibrary.com]

Meanwhile, she came to the emergency room when she noticed that her speech was impaired. She was unaware of the slight weakness in her left arm and hand. On examination, mild dysarthria and moderate left hemiparesis were observed. Her neurological findings lasted approximately 36 h. After that, doppelgänger, the sense of unfamiliarity of the left hand, and left hemiparesis completely resolved. In the comprehensive neuropsychological examination of the patient, mild anxiety and restlessness were detected, except for a few errors and deficiencies in attention and episodic and semantic memory tasks. The patient had no signs of visuospatial neglect (Table 1). Diffusion‐weighted brain MRI revealed a right small superior parietal lobule (SPL) ischemic lesion extending to the precuneus (Figure 1). electroencephalography (EEG) was normal at the first week of stroke. Neurovascular examination did not reveal a specific stroke etiology.

**Table 1:** Demographic, clinical, and neuropsychological findings

Age/sex	Locus of lesion	Tactile size, shape, texture discrimination	Autotopagnosia for body parts	Autotopagnosia for sensory sensations	Anosognosia for hemiparesis	Somatoparaphrenia	Fading limb	Tactile extinction	Right–left discrimination	Finger recognition	Psychiatric comorbidity	Cause of stroke
1/62/F	SPL+ precuneus	+	−	−	+	+	−	−	−	−	None	Unknown
2/65/M	SPL	−	−	−	−	−	+	+	−	+	None	Atrial fibrillation
3/57/M	SPL+ precuneus	+	+	+	−	−	−	−	+	−	None	Carotid artery dissection

Abbreviations: A, arm; B, brachial; F, face; F, female; L, leg; M, male; SPL, superior parietal lobule; T, trunk.

### 2.2 CASE 2

A right‐handed 65‐year‐old retired man without neurological or psychiatric disease had a history of a strange feeling of his left upper side and difficulty in controlling his arm and hand during a movement. Meanwhile, he felt that the image of a person [his own face] on his left side was first about 50 cm in front of him, and this continued 2–3 times at 15 min intervals. He could identify the face and upper part of the body, including the clothing and facial expression, of his companion. The image always appeared on the left side and in front of it and disappeared when the patient closed his eyes. He also thought that someone was making fun of him when his dressing, movements, sitting, and standing were the same. At hospital admission, he had mild weakness on his left side. He could not use his left hand when it was not under visual control and could not mentally represent and locate this part of the body in space (eyes closed), with an accompanying feeling of transparency. Additionally, at rest, the patient had difficulties in locating his left hand in space when it was not seen (Table 1). His weakness and body image disorder resolved within 24 h. In his neuropsychological examination, there were nonsignificant delays and errors in the Stroop test and working memory tests. Diffusion‐weighted brain MRI revealed a small right SPL ischemic lesion (Figure 1). EEG on admission was normal. Atrial fibrillation was found during cardiac examination.

### 2.3 CASE 3

A 57‐year‐old right‐handed male patient suddenly felt numbness and tingling in his left hand and foot and developed clumsiness when using this side. The patient was sitting at the table and trying to figure out what happened, when another “me” presented next to him and seemed to want to talk to him. He said «this is my double». He was doing the moves before him and trying to make him feel like he should do the same moves. This familiar person did not say a word. He always wanted to do something. He repeated every move of his, in a way that made him angry. When he got into bed, the man in the room also went to bed. He felt that he was lying there, and the double was lying parallel to him. In these moments, he truly believed that he and his double were real. When he went to the hospital with these complaints, he showed inability to locate tactile stimuli when they were applied below the contralesional elbow. All stimulations were experienced as being applied at the level of the elbow and knee (verbally or by pointing with eyes closed), while they had actually been applied to the hand and leg. In other locations, the patient was very precise when stimulations were applied both on the contralesional upper and on the lower body (Table 1). MRI revealed an ischemic lesion in the right SPL and precuneus. His autotopagnosia for sensory sensations was recovered rapidly within 48 h. EEG at admission and at a later period was normal. Magnetic resonance angiography revealed a crescent‐shaped intramural hematoma in the right internal carotid artery adjacent to the vessel lumen, suggesting artery dissection.

## 3 DISCUSSION

Our patients represent unreported cases in terms of showing autoscopic phenomena such as FOP and BIBS disorders. (i) A sense of presence (FOP, doppelgänger) and different BIBS disorders were present in all cases. (ii) The first patient was considered to have a combination of FOP and somatoparaphrenia, the second patient had simultaneous FOP and fading limb, and the third patient had concomitant FOP and autotopagnosia for sensory sensations. (iii) These reduplication syndromes and BIBS disorders developed due to a lesion in the superior posterior parietal lobule (SPL) extending to the precuneus, where the body schema organization was established. The combination of autoscopic phenomena and BIBS, the “reduplication and the body image disorder,” is extremely rare but not unique. Our first case is very similar to the case of coexistence of FOP and somatoparaphrenia (delusional elaboration of disownership) following right lower parietal lobule hemorrhage reported by Pötzl in 1925.^11^ The findings of fading limb in the second patient with impaired ability to localize a limb in space and autotopagnosia in the third patient with reduced awareness of parts of the body suggested involvement of the parietal area, which provides body image and schema functions in humans. This is the “classic” lesion site for BIBS disorders.^12^ However, in addition to parietal or parieto‐temporal pathology, the spread of the lesion to the frontal‐opercular areas may alter the clinical picture; orbitofrontal lesion extension may yield delusional elaboration of disownership, and latero‐frontal propagation is more commonly associated with asomatognosia.^13^ Parietal involvement in all cases is a necessary but not sufficient condition for the occurrence of BIBS disorders. It appears that a person's sense of self occurs through the operation of the frontoparietal network. For this reason, the parietal lobes can be considered as the integration center of both the perception of the body and the imagination of the structure of the body and combining them with the self. Moreover, to the best of our knowledge, FOP and either fading limb or autopognosia in combination have never been reported after an SPL and precuneal lesion.

The syndrome most frequently discussed in connection with the autoscopic phenomena is Capgras syndrome, which can be seen in dementias. Its main symptom is the belief that someone else has a twin and this couple pretends to be fake replaces the original. In fact, there are a number of fundamental differences between them. First, the autoscopic phenomenon always includes a sensory‐perceptual element (i.e., the person's twin is seen and/or felt). Conversely, in misidentification/duplication syndromes, only the existence of the doppelgänger is claimed and no specific physical location of the doppelgänger is mentioned. Second, autoscopic replication lacks the element of misidentification and insight is typically preserved. In conditions such as Alzheimer's dementia, the person's sense of self is erased, but the autoscopic double is one's real self, while in dementia misidentification syndromes, the doppelgänger is always experienced as an impostor or stranger.

Vascular lesions, tumors, and encephalitis are on the extensive causal list for BIBS, although studies on FOP have focused on focal or diffuse cerebral diseases.^1^, ^2^, ^7^ Acute vascular lesions involving a parietal lobe were mostly associated with BIBS disorders,^14^ while FOP is rarely presented with a circumscribed cerebral pathology due to acute vascular lesions. Other patients with larger and extensive even bilateral SPL and precuneus lesions have not reported these symptoms.^15^ In our registry, there are no other cases with the same lesion and no such symptoms to compare the side and extent of the lesions.

The medial portion of this area is supplied by the anterior cerebral artery, while the lateral portion is supplied by the middle cerebral artery. Therefore, it is extremely rare to find such isolated superior parietal lobule infarcts. As the lesions were quite small, the symptoms of the patients disappeared within 2 days.

## 4 CONCLUSION

All patients reported both the invisible doppelgänger presence and at least one sense of disorder of the BIBS. BIBS and FOP disorders may coexist as a result of SPL and precuneus lesions, which are involved in self‐centered mental body imagery processing.

## AUTHOR CONTRIBUTIONS

Emre Kumral is the principal author. Emre Kumral and Fatma E. Çetin were involved in developing the study concept and design. Birgül Dere was involved intheacquisition of data. Fatma E. Çetin and Hüseyin N. Özdemir were involved in analysis and interpretation of data. Emre Kumral was involved in study supervision and coordination.

## CONFLICT OF INTEREST

The authors declare no conflict of interest.

## ETHICS STATEMENT

The experiments involving the subjects were approved by the Medical Ethics Committee of Ege University and were carried out according to the Declaration of Helsinki (approval number: 22‐5T/59).

## Data Availability

The data sets used and/or analyzed during the current study are available from the corresponding author on reasonable request.
